# Ileostomy Complications in Infants less than 1500 grams – Frequent but Manageable

**DOI:** 10.21699/jns.v6i1.451

**Published:** 2017-01-01

**Authors:** Simon Kargl, Oliver Wagner, Wolfgang Pumberger

**Affiliations:** 1 Department of Pediatric Surgery, Kepler University Hospital, Linz; 2 Department of Neonatology, Kepler University Hospital, Linz

**Keywords:** Ileostomy, Stoma, Very low birth weight infants, Complications

## Abstract

Background: In very low birth weight infants abdominal emergency surgery may result in ileostomy formation. We observed a frequent stoma complications in these patients. This retrospective analysis put light on ileostomy-related problems and complications in very low birth weight (VLBW) infants.

Materials and Methods: In a seven-year retrospective chart review (2008 - 2014) infants with ileostomy formation weighing less than 1500 grams at time of operation were identified and reviewed. Data analysis included demographic data, complications and short term outcomes.

Results: Thirty patients were included. Ileostomy was formed for spontaneous intestinal perforation (SIP) (n=17), meconium obstruction of prematurity (MOP) (n=6), midgut volvulus (MV) (n=5), necrotizing enterocolitis (NEC) (n=1) and Hirschsprung’s disease (HD) (n=1). Three patients died before ileostomy reversal was considered. In seven patients planned ileostomy reversal was done. Twenty infants had stoma related complications (stoma prolapse, prestomal obstruction, stoma retraction, high output stoma, peristomal skin excoriation, and stomal ischemia). Complications did not correlate with underlying diseases. Stomal complications necessitated earlier stoma reversal (mean 62 days). Postoperative complications after stoma reversal occurred in three children (wound dehiscence, adhesion ileus, anastomotic stricture).

Conclusions: Although ileostomy related complications are frequent in very low birth weight infants, mortality is low. Morbidity is manageable.

## INTRODUCTION

In VLBW infants with intestinal perforation, necro-sis or obstruction, ileostomy formation can be necessary or even lifesaving. Ileostomy can be cre-ated safely and in a short time even in VLBW in-fants but ostomy related problems frequently occur in these patients. Our study focused on problems and complications of ileostomy formation and its closure in infants weighing less than 1500g.

## MATERIALS AND METHODS

This is a seven-year retrospective medical chart review. From 2008 to 2014 all patients with ileos-tomy formation were selected from database. In-fants with a bodyweight less than 1500 grams at the time of surgery were included. Infants with jeju-nostomy, colostomy or multiple stomas were ex-cluded. Patients were divided in two groups depend-ing on the presence or absence of stomal complica-tions. Data was collected and compared including age and weight at surgery, underlying disease, type of surgery as well as ostomy related problems, tim-ing and indication of ostomy closure.

## RESULTS

We identified 30 infants who met the inclusion criteria (15 male/15 female). All children were born prematurely, from 24+1 to 32+0 weeks of gestation (median 25+5). Ileostomy at the level of distal ileum was formed for SIP in 17 patients, MV in 5 patients, MOP in 6 patients, HD in 1 patient, and NEC in 1 patient. In 26 of 30 cases a double-barreled ileostomy was performed. In 25 of these 26 patients, the proximal and distal limbs were exteriorized through a separate incision in the right lower quadrant. In one case the stomas were brought out through the laparotomy incision (Supra-umbilical right transverse). In three cases an ileal end stoma and in one case loop ileostomy were formed. Stoma bags were used in all cases in postoperative period.

At time of ileostomy formation mean age was 18 days (range 3-99 days) and mean weight was 887 grams (range 510-1480 grams). The mean duration of surgery for ileostomy formation was 49 minutes (range: 36-74 min). In the early postoperative period, one neonate with SIP died of fatal liver hemorrhage after few hours of forming an ileostomy. Two other patients died of concomitant diseases of prematurity before reversal of stoma. Seven patients were discharged from hospital between ileostomy creation and closure (3 from cohort with no stoma related complications and 4 from cohort with stoma related complications).

In seven patients (cohort with no stoma related complications), ileostomy closure was performed at a weight of about 2000 grams and/or a significantly improved overall condition. Ostomy reversal was carried out without postoperative problems after a mean period of 97 days (range 42-149).

In 20 infants (cohort with stoma related complications) stomal complications necessitated earlier closure after a mean period of 62 days (range 11-149). The following complications preponed stoma closure: peristomal skin excoriation, stoma prolapse, high output stoma (> 20 mls/kg/day plus poor weight gain), stoma retraction, prestomal obstruction and ischemia (Table 1). None of these stomal complications proved fatal.

The occurrence of stomal complications did not correlate with underlying diseases or with the type of ileostomy. At time of surgery, the group of patients with stomal complications showed a younger age and a lower weight (statistically insignificant) (Table 2). In patients with stomal complications ileostomy reversal was done significantly earlier (Fig.1).

Twenty-four patients had a routine contrast study prior to ileostomy closure (distal loopogram or contrast enema) to rule out colonic stricture (n=0). In three patients with complications (1 necrosis/ischemia and 2 with prestomal strangulation) distal cologram was not performed to avoid delay in surgical treatment. Intestinal reconstruction was performed via limited peristomal incision in all seven cases of planned ileostomy closure and in two cases of early closure due to stomal retraction. Relaparotomy was necessary in the remaining eighteen patients with stomal complications. Mean operation time was 110 minutes (range: 63 - 138). Of 27 ileostomy closure procedures, three patients (11%) suffered from postoperative complications necessitating reoperation: one early complication (wound dehiscence) and two late complications (adhesion ileus, 1 anastomotic stricture). These three patients were from the stomal complications cohort (15%; 3 out of 20). In all patients of group 1, ileostomy closure was done without any postoperative complications. No anastomosis dehisced and short-term morbidity was 0% due to ileostomy closure.

**Figure:**
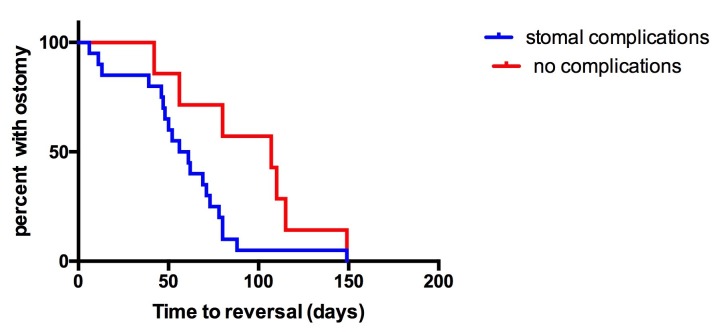
Figure 1: Kaplan-Meier curve of number of patients with ileostomy. In complication group an early reversal is evident.

**Figure:**
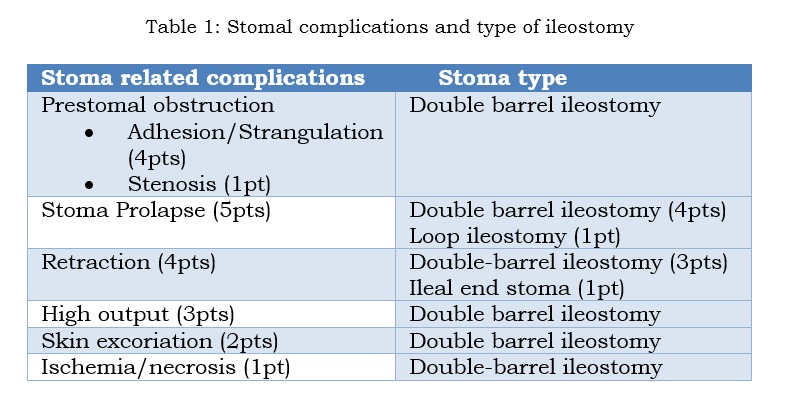
Table 1: Stomal complications and type of ileostomy

**Figure:**
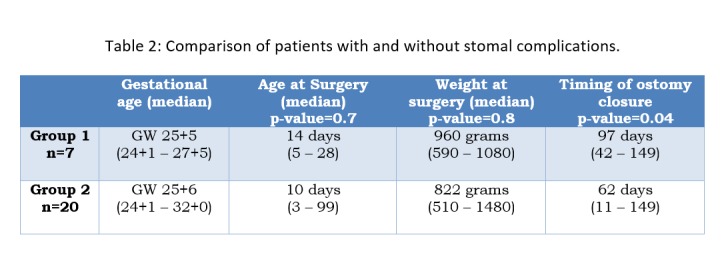
Table 2: Comparison of patients with and without stomal complications.

## DISCUSSION

The optimal surgical management of VLBW infants with neonatal bowel perforation is a matter of discussion [1]. Nevertheless, ileostomy formation is an important strategy in abdominal emergency surgery in these children. Different techniques are used and there is still a debate whether to use a separate incision for ostomy or not [2]. It remains unclear which type of ostomy is superior and may lead to lower incidence of stoma-related complications [3]. In VLBW infants we usually perform a Mikulicz type double-barrel ileostomy brought out through a separate incision in the right lower quadrant. This placement is suitable for good stomal care avoiding the skin creases (groin and umbilicus) and bony prominences (superior anterior iliac spine). In one patient, we created an ileostomy through the laparotomy wound which worked well without any demerits.

Ileostomy creation is a safe procedure, even in VLBW infants [4,5] and can be performed in a less time, which may be important to avoid major temperature and fluid imbalances [6]. In VLBW infants, postoperative deaths are due to sepsis, progressive abdominal catastrophes and concomitant diseases of prematurity [7,8]. In our series one infant died in the early postoperative period because of fatal liver hemorrhage, a feared complication associated with laparotomy in VLBW infants [9].

Ileostomy complications seem to be very frequent in VLBW infants [10-12]. The high rate of stoma related complications in VLBW infants in different series suggests that morbidity of an ileostomy is inherent in the procedure and not necessarily the result of the way it is constructed [3, 10-12].

It is interesting that although the anterior abdominal wall is weak in VLBW infants, we had not a single case of parastomal hernia. Aguayo et al. reported 42% stoma-related complications in neonates with necrotizing enterocolitis [10]. They conclude that premature infants carry a risk for developing stoma-related complications. In a retrospective review of premature infants with NEC, O´Connor et al. found that 68% of infants treated with ostomy formation developed complications [11]. They conclude that these findings argue for primary anastomosis. Although we had a similar high rate of stomal complications, we do not draw this conclusion. As shown in our series stomal complications were not fatal; nonetheless, necessitated early unplanned reversal of the ileostomy.

There is no general recommendation concerning timing of ostomy closure in neonates but it has been shown that early ileostomy closure can be done safely in neonates [13-15]. In our experience, most stomal complications e.g. stomal prolapse or skin excoriation evolve with time. In the complications group ostomy closure was done after a mean period of 2 month - earlier closure might have reduced the number of stomal complications significantly (Fig.1).

We are aware of the limitations of our study due to the small number of patients. Nevertheless, despite of the high incidence of stomal complications the mortality and short-term morbidity of both procedures: ileostomy formation and ileostomy reversal seems tolerable in VLBW infants.

To conclude, ileostomy formation may be a lifesaving procedure in VLBW infants. Ileostomy related complications in VLBW infants are frequent but manageable.

## Footnotes

**Source of Support:** None

**Conflict of Interest:** None
